# circRNA_141539 can serve as an oncogenic factor in esophageal squamous cell carcinoma by sponging miR-4469 and activating CDK3 gene

**DOI:** 10.18632/aging.103071

**Published:** 2021-01-27

**Authors:** Zheng-hua Liu, Shi-ze Yang, Wen-ya Li, Si-yuan Dong, Si-yu Zhou, Shun Xu

**Affiliations:** 1 Department of Thoracic Surgery, The First Affiliated Hospital of China Medical University, He-Ping, Shen-Yang 110001, Liao-Ning Province, China

**Keywords:** ESCC, circRNA_141539, miR-4469, CDK3, progression

## Abstract

The abnormal expression and regulation of circular RNA (circRNA) is involved in the occurrence and development of a variety of tumors. The current study aimed to determine the role of circRNA_141539 in esophageal squamous cell carcinoma (ESCC). CircRNA_141539 expression in ESCC was detected via circRNA chip analysis and verified via reverse transcription-quantitative PCR. Associations between circRNA_141539, patient clinicopathological characteristics and prognosis were also statistically analyzed. Additionally, the effects of circRNA_141539 on ESCC cell proliferation and invasion were assessed. A dual-luciferase assay was performed to analyze the interaction between circRNAs, microRNAs (miRs) and mRNAs. The results revealed that circRNA_141539 was significantly up-regulated in patients with ESCC. Furthermore, high circRNA_141539 expressions were significantly associated with TNM stage, differentiation and poor prognosis, revealing high diagnostic value (P<0.05). Furthermore, circRNA_141539 overexpression promoted cell proliferation and invasion, while circRNA_141539 silencing inhibited cell proliferation and invasion (P<0.05). The dual-luciferase reporter assay identified that circRNA_141539 directly binds to miR-4469 and also revealed that cyclin-dependent kinase-3 (CDK3) was negatively regulated by miR-4469. The results indicated that circRNA_141539 served as an oncogenic factor in ESCC by sponging miR-4469 and activating CDK3 expression. circRNA_141539 may present as a novel diagnostic and prognostic biomarker and a therapeutic target for patients with ESCC.

## INTRODUCTION

Esophageal cancer (EC) is one of the most common malignant tumors of the digestive tract, representing a serious threat to human health and life [1]. Esophageal squamous cell carcinoma (ESCC) is the major pathological type of EC, accounting for ~85% of all patients [2]. Radical surgery, radiotherapy and chemotherapy are the main treatments for EC [3, 4]. However, the overall prognosis of patients with EC remains poor and the infiltration and metastasis of this tumor is the major cause of treatment failure and mortality [5].

Non-coding RNA (ncRNA) is a type of RNA that serves various regulatory functions [6]. Circular RNA (circRNA) is a novel class of endogenous ncRNA with covalently linked 3’- and 5’-ends, creating a closed-loop structure that is not easily degraded by exonucleases [7]. The abnormal expression of circRNAs is observed in various tumors, suggesting that circRNAs are involved in tumorigenesis and cancer progression [8]. Recent studies have revealed that circRNA serves a regulatory role by competitively binding to miRNA, ultimately affecting target gene expression [9]. CircRNAs are considered to be one of the most ideal and specific biomarkers for patient diagnosis and prognosis in various types of cancer [10].

The present study analyzed the expression profile of circRNAs and identified that circRNA_141539 was significantly up-regulated in ESCC tissue. The results also revealed that circRNA_141539 served as a miR-4469 sponge to activate cyclin-dependent kinase-3 (CDK3) expression and consequently promote ESCC progression. It was concluded that circRNA_141539 may present a novel diagnostic and prognostic biomarker and therapeutic target for patients with ESCC.

## RESULTS

### circRNA_141539 is significantly upregulated in ESCC tissues

**Figure 1:**
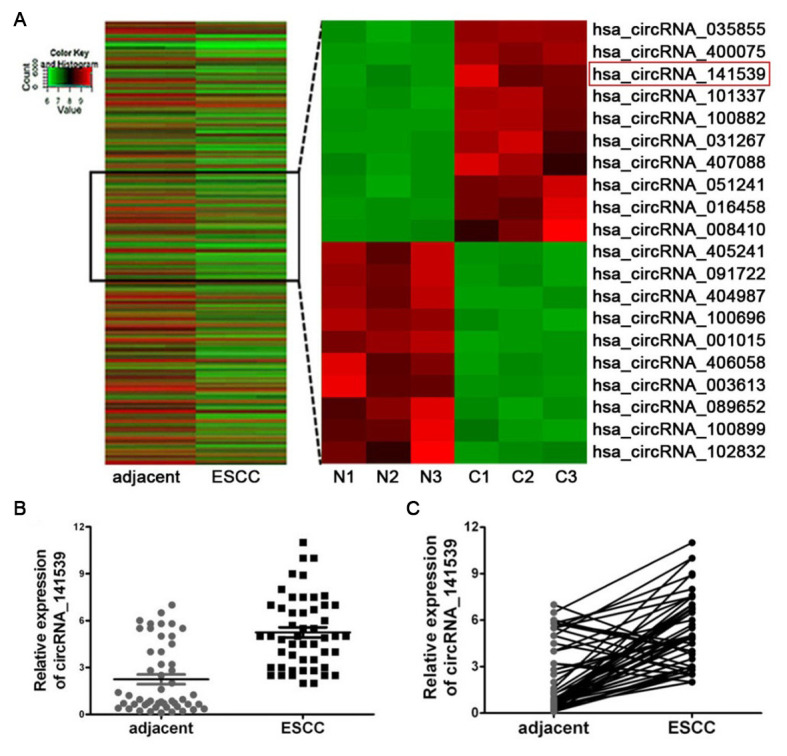
**circRNA_141539 was up-regulated in ESCC tissue.** (**A**) Hierarchical clustering analysis showed the top 10 most up-regulated and down-regulated circRNAs. The expression levels are presented in different colors indicating expression levels. (**B**, **C**) qRT-PCR assay showed that circRNA_001937 was significantly up-regulated in ESCC tissues (n=50), ^*^P < 0.05, ^**^P < 0.01 versus corresponding adjacent tissues. circRNA_141539 expression levels were normalized to GAPDH. N: adjacent normal tissues, C: ESCC tissues.

CicrRNA expression was determined using Arraystar Human circRNAs chips with three paired ESCC and adjacent non-cancer tissues. Following normalization and data analysis, a total of 87 significantly up-regulated circRNAs and 73 significantly down-regulated circRNAs were revealed. A cluster heat map was subsequently constructed, which listed the top 10 most up-regulated and down-regulated circRNAs (Figure 1A; P<0.05). The details of top 10 up-regulated and down-regulated differentially expressed circRNAs are listed in Supplementary Table 1. Among the 30 differently expressed circRNAs, circRNA_141539 was 7.58-fold up-regulated in ESCC tissues. RT-qPCR was then performed to detect the expression of circRNA_141539 in 50 paired ESCC and adjacent tissues. The results confirmed that circRNA_141539 was significantly up-regulated in ESCC tissues (n=50; Figure 1B, 1C; P <0.05) compared with corresponding adjacent non-cancer tissues.

### circRNA_141539 is associated with patient diagnosis and prognosis in ESCC

**Figure 2:**
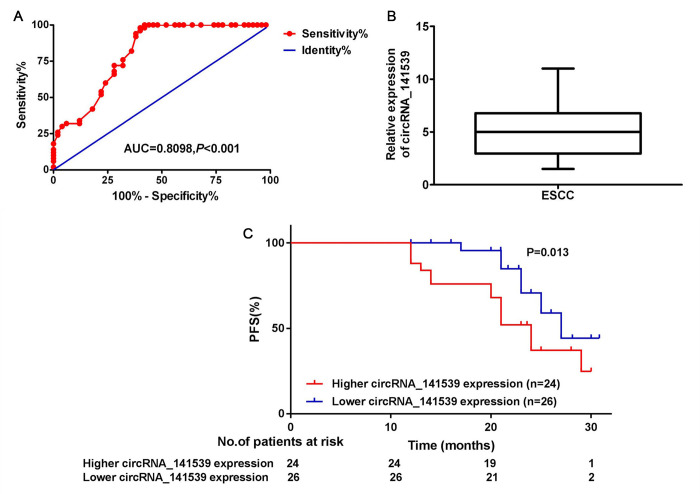
**circRNA_141539 was associated with diagnosis and prognosis in ESCC.** (**A**) The median expression of circRNA_141539. (**B**) ROC curve showed the diagnostic efficiency of circRNA_141539. (**C**) Kaplan- Meier progression-free survival curves showed the association between high and low circRNA_141539 expression.

A receiver operating characteristic curve was constructed to assess the diagnostic value of circRNA_141539 in ESCC. The results revealed that circRNA_141539 expression had high diagnostic efficiency, with an area under the curve value of 0.8098 (95% confidence interval, 0.6874-1.254) in 50 patients with ESCC (Figure 2A; P<0.05). Subsequently, the 50 cases of ESCC were divided into high or low circRNA_141539 expression groups using the median circRNA_141539 expression value as a cutoff (Figure 2B). Progression-free survival (PFS) curves were constructed using the Kaplan-Meier method and log-rank tests. The results revealed that patients with high circRNA_141539 expressions exhibited significantly shorter PFSs ((mPFS, 21.5 mo. vs. 26.6 mo.; P=0.013; Figure 2C) than those with low circRNA_141539 expressions. Furthermore, multivariate Cox regression analysis revealed that high circRNA_141539 expressions along with low differentiation and stage III were poor survival prognostic factors for patients with ESCC (Table 1; P<0.05). These data indicated that circRNA_141539 serves as a diagnostic and prognostic biomarker for patients with ESCC.

**Table 1:** Multivariate analyses for overall survival by Cox regression test.

**Characteristic**	**OR (95%CI)**	**p**
Age ≥ 60y	0.95(0.68-1.75)	0.685
Gender	1.20(0.82-2.52)	0.538
Pathological grade		
Low	1.82(1.51-2.23)	0.035
High/middle	Reference	
Cancer stage		
Stage III	1.88(1.01-2.52)	0.045
Stage I/II	Reference	
High circRNA_141539 expressions	1.58(1.06-2.45)	0.023

OR, Odds Ratio; CI, confidence interval.

### circRNA_141539 is associated with clinicopathological characteristics in patients with ESCC

**Figure 3:**
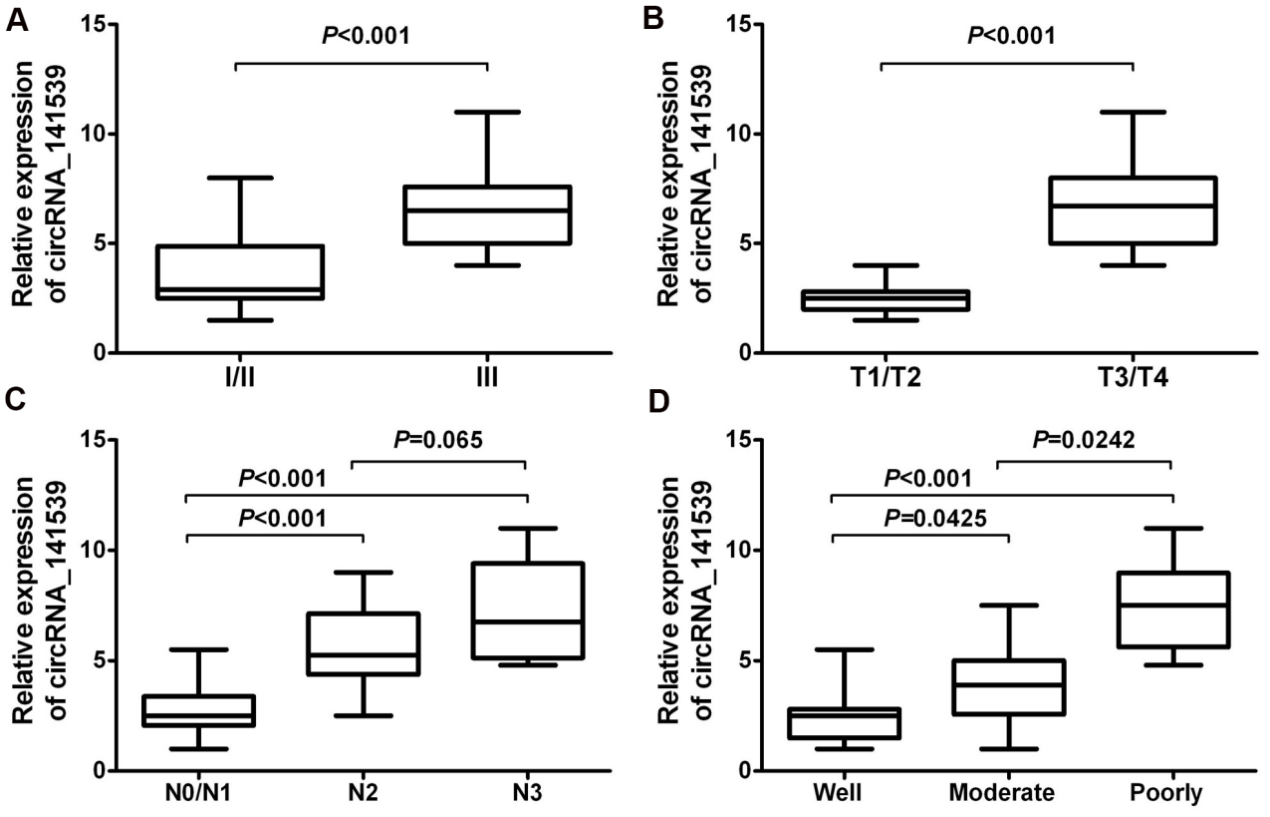
**circRNA_141539 was associated with clinicopathological characteristics in ESCC.** (**A**) The association of circRNA_141539 with TNM stage, (**B**) with T stage, (**C**) with N stage, and (**D**) with histological grade.

A chi-square test was employed to estimate the relationship between circRNA_141539 expression and the clinicopathological characteristics of patients with ESCC. The results demonstrated that higher circRNA_141539 expressions were positively associated with TNM stage (Figure 3A; P<0.001), T stage (Figure 3B; P<0.001) and N stage (Figure 3C; P<0.001), and negatively associated with histological grade (Figure 3D; P<0.001). However, no significant association between N2 and N3 stages was identified (Figure 3C; P=0.065).

### circRNA_141539 promotes cell proliferation and invasion

**Figure 4:**
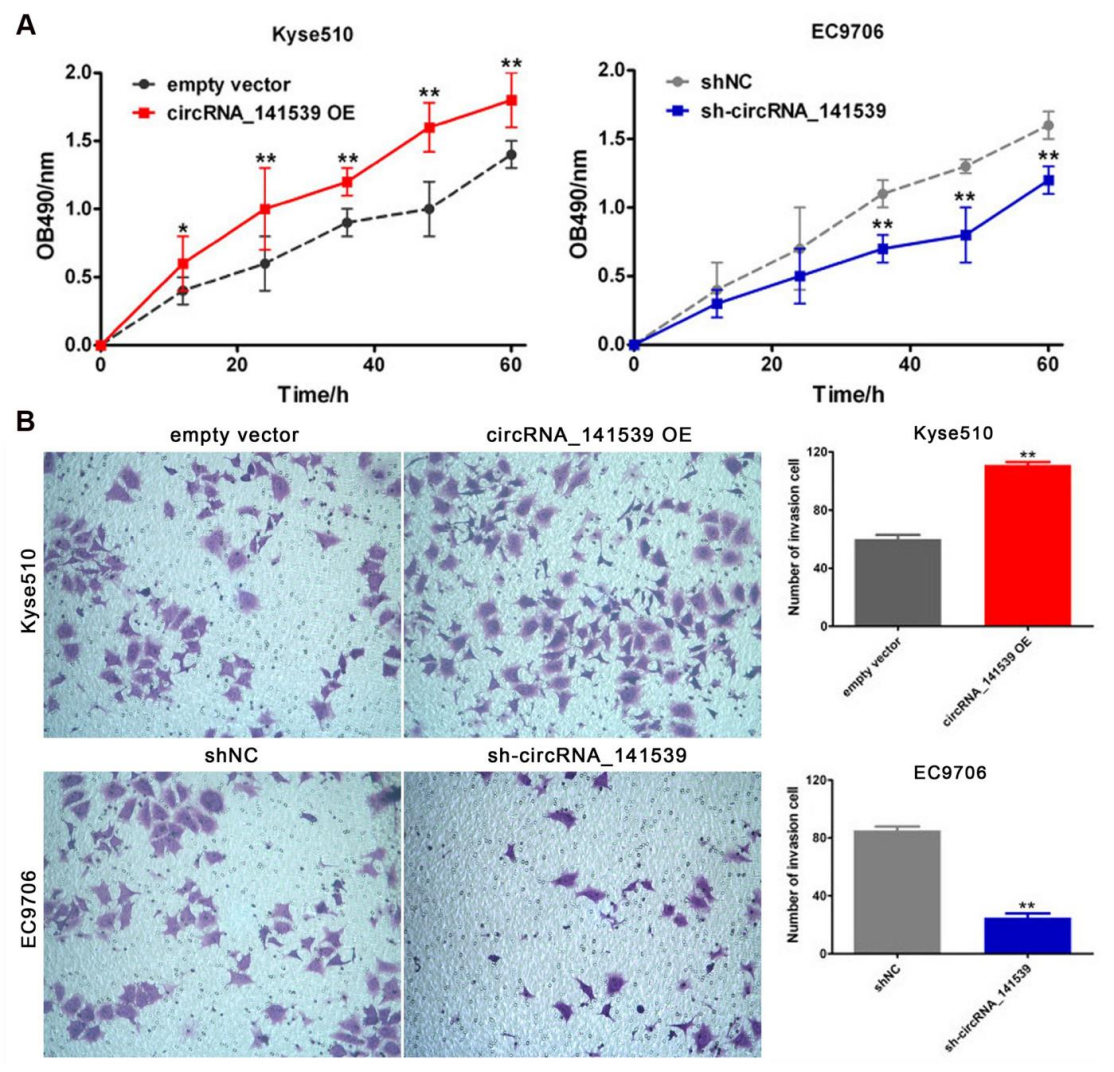
**circRNA_141539 can promote cell proliferation and invasion (n=3).** (**A**) MTT assay; (**B**) Transwell chamber invasion assay. ^*^P < 0.05, ^**^P < 0.01 versus corresponding NC group.

The current study performed RT-qPCR to evaluate the expression of circRNA_141539 in human ESCC cell lines. The results revealed that circRNA_141539 expression was highest in EC9706 cells and lowest in Kyse510 cells when compared with Het-1A cells (Supplementary Figure 1A; P<0.05). The circRNA_141539 OE vector was transfected into Kyse510 cells to stably up-regulate the expression of circRNA_141539. Additionally, shRNA was transfected into EC9706 cells to stably silence the expression of circRNA_141539. Transfection efficiency was verified via RT-qPCR (Supplementary Figure 1B, 1C; P<0.05). The effect of circRNA_141539 on cell proliferation and invasion were subsequently evaluated via MTT and Transwell invasion assays. The results revealed that circRNA_141539 overexpression promoted Kyse510 cell proliferation and invasion, while circRNA_141539 silencing inhibited EC9706 cell proliferation and invasion (Figure 4A, 4B: P<0.05).

### circRNA_141539 acts as a miR-4469 sponge

**Figure 5:**
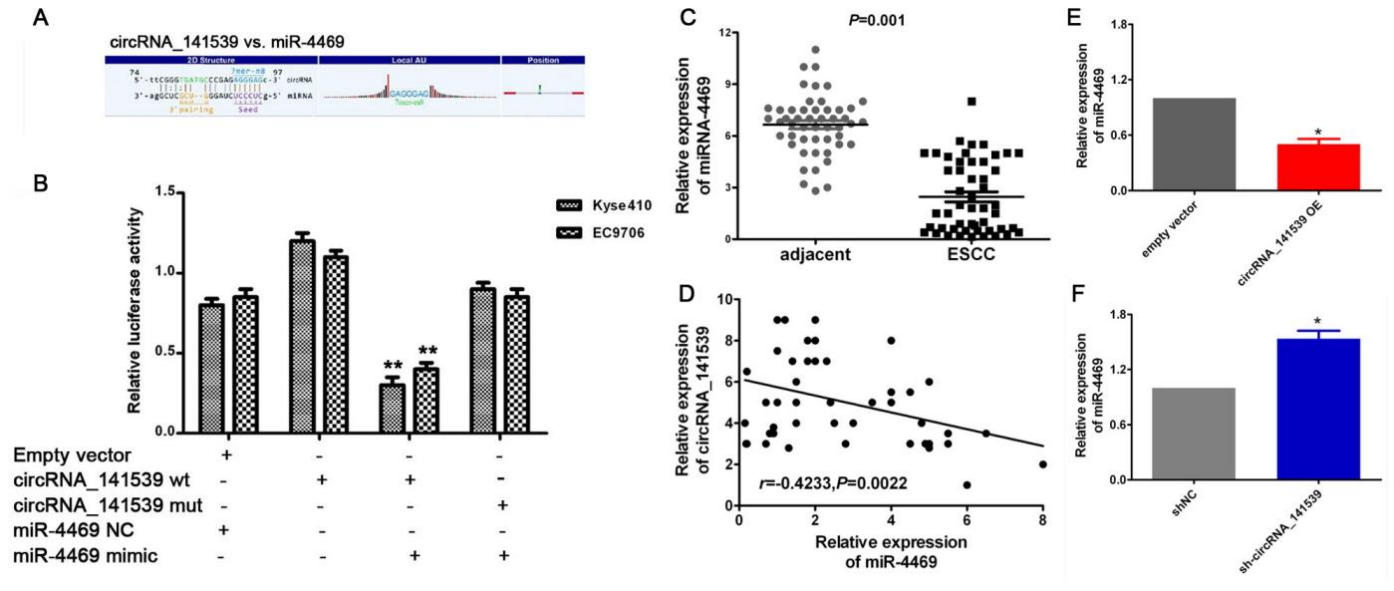
**circRNA_141539 acts as a miRNA sponge for miR-4469.** (**A**) Schematic diagram showed that miR-4469 specifically binds to 3′-UTR of circRNA_141539. (**B**) Dual-luciferase assay results. (**C**) qRT-PCR showed miR-4469 was were significantly down-regulated in ESCC tissues (n=50), ^*^P < 0.05, ^**^P < 0.01 versus corresponding adjacent tissues. (**D**) Pearson's correlation showed that circRNA_141539 was negatively correlated to miR-4469 expression. (**E**, **F**) qRT-PCR showed miR-4469 were significantly decreased in circRNA_141539 OE group, and dramatically increased in sh-circRNA_141539 group. ^*^P < 0.05, ^**^P < 0.01 versus corresponding NC group.

A dual-luciferase reporter assay was performed to verify the MRE-based circRNA_141539-miR-4469 interaction. A schematic diagram constructed using Arraystar homemade software revealed that miR-4469 binds to the wild-type sequence of circRNA_141539 (Figure 5A). Dual- luciferase assays results also demonstrated that relative luciferase activity in the psiCHECK2-circRNA_141539-wt and miR-4469 mimic co-transfection groups was significantly lower compared with the miR-4469 NC or psiCHECK2- circRNA_ 141539-mut groups. However, no significant differences were identified in the psiCHECK2-circRNA_141539-mut group compared with the miR-4469 mimics and miR-4469 NC groups (Figure 5B; P<0.05). The results of RT-qPCR demonstrated that miR-4469 was significantly down-regulated in ESCC tissues (n=50) compared with corresponding adjacent tissues (Figure 5C; P<0.05). Furthermore, Pearson's analysis revealed that circRNA_141539 was negatively correlated with miR-4469 expression (Figure 5D; P<0.05). miR-4469 expression was also determined to be significantly and negatively regulated by circRNA_141539 expression in Kyse510 and EC9706 cells (Figure 5E, 5F; P<0.05).

### CDK3 is a direct target of miR-4469

**Figure 6:**
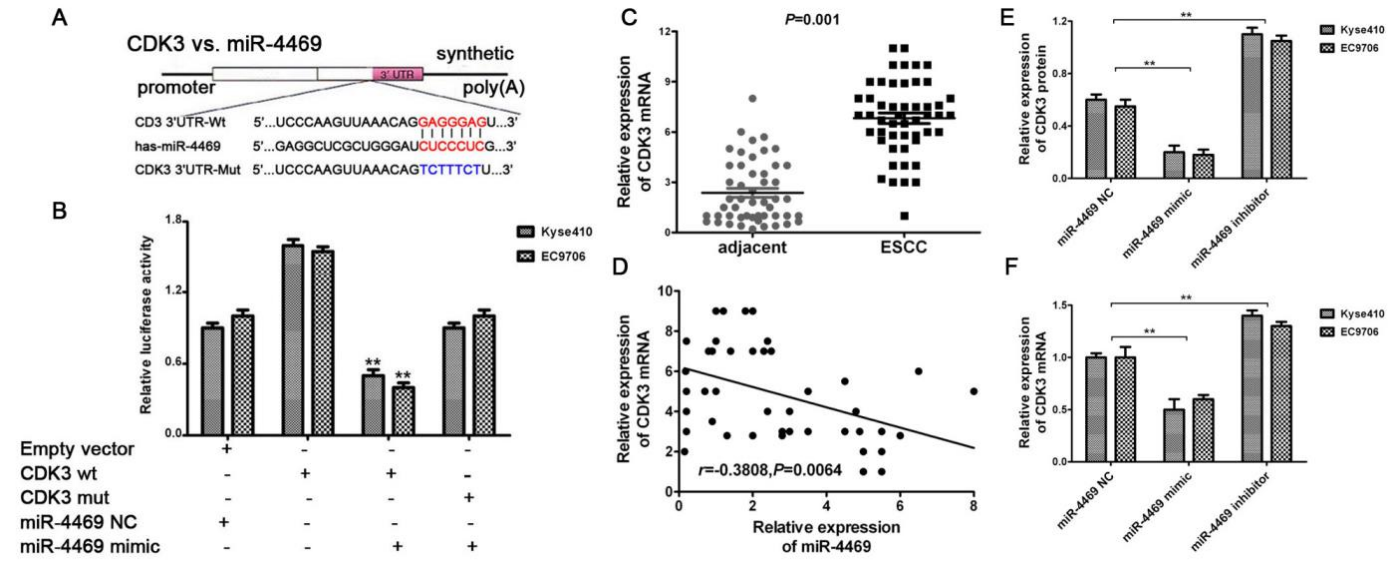
**CDK3 was identified as a direct target of miR-4469.** (**A**) Schematic diagram showed that miR-4469 specifically binds to the wild-type 3’-UTR sequence of CDK3. (**B**) Dual-luciferase assay results. (**C**) qRT-PCR results showed that CDK3 mRNA was significantly up-regulated in ESCC tissues (n=50), ^*^P < 0.05, ^**^P < 0.01 versus corresponding adjacent tissues. (**D**) Pearson's correlation showed that CDK3 mRNA was negatively correlated to miR-4469 expression. (**E**) Western blot and (**F**) qRT-PCR results showed that the expression of CDK3 proteins and mRNA were significantly decreased in miR-4469 mimic group, and dramatically increased in miR-4469 inhibitor group. ^*^P < 0.05, ^**^P < 0.01 versus miR-4469 NC group.

The miR-4469 mimic and inhibitor were transfected into Kyse510 and EC9706 cells. RT-qPCR showed that miR-4469 was significantly increased in the mimic group, and reduced in inhibitor group, compared with the group (Supplementary Figure 2A, 2B, both P<0.05).Dual-luciferase reporter assay was performed to determine whether CDK3 was a direct target of miR-4469. The schematic diagram revealed that miR-4469 specifically binds to the Wt 3’-UTR sequence of CDK3 (Figure 6A). The results also demonstrated that relative luciferase activity in the psiCHECK2-CDK3-3'-UTR-wt and miR-4469 mimic co-transfection group was significantly lower compared with miR-4469NC or psiCHECK2-CDK3-3'-UTR-mut groups. However, no significant differences were identified in the psiCHECK2- CDK3-3'-UTR-mut group compared with the miR-4469 mimics and miR-4469 NC groups (Figure 6B; P<0.05). Additionally, the results of RT-qPCR indicated that CDK3 mRNA was significantly up-regulated in ESCC tissues (n=50) compared with corresponding adjacent tissues (Figure 6C; P<0.05). Pearson's correlation analysis also demonstrated that CDK3 mRNA was negatively correlated with miR-4469 expression (Figure 6D; P<0.05). Furthermore, western blotting and RT-qPCR results confirmed that CDK3 protein and mRNA expression were significantly and negatively regulated by miR-4469 in Kyse510 and EC9706 cells (Figure 6E, 6F; P<0.05).

### miR-4469 and CDK3 are associated with clinicopathological characteristics in ESCC

**Figure 7:**
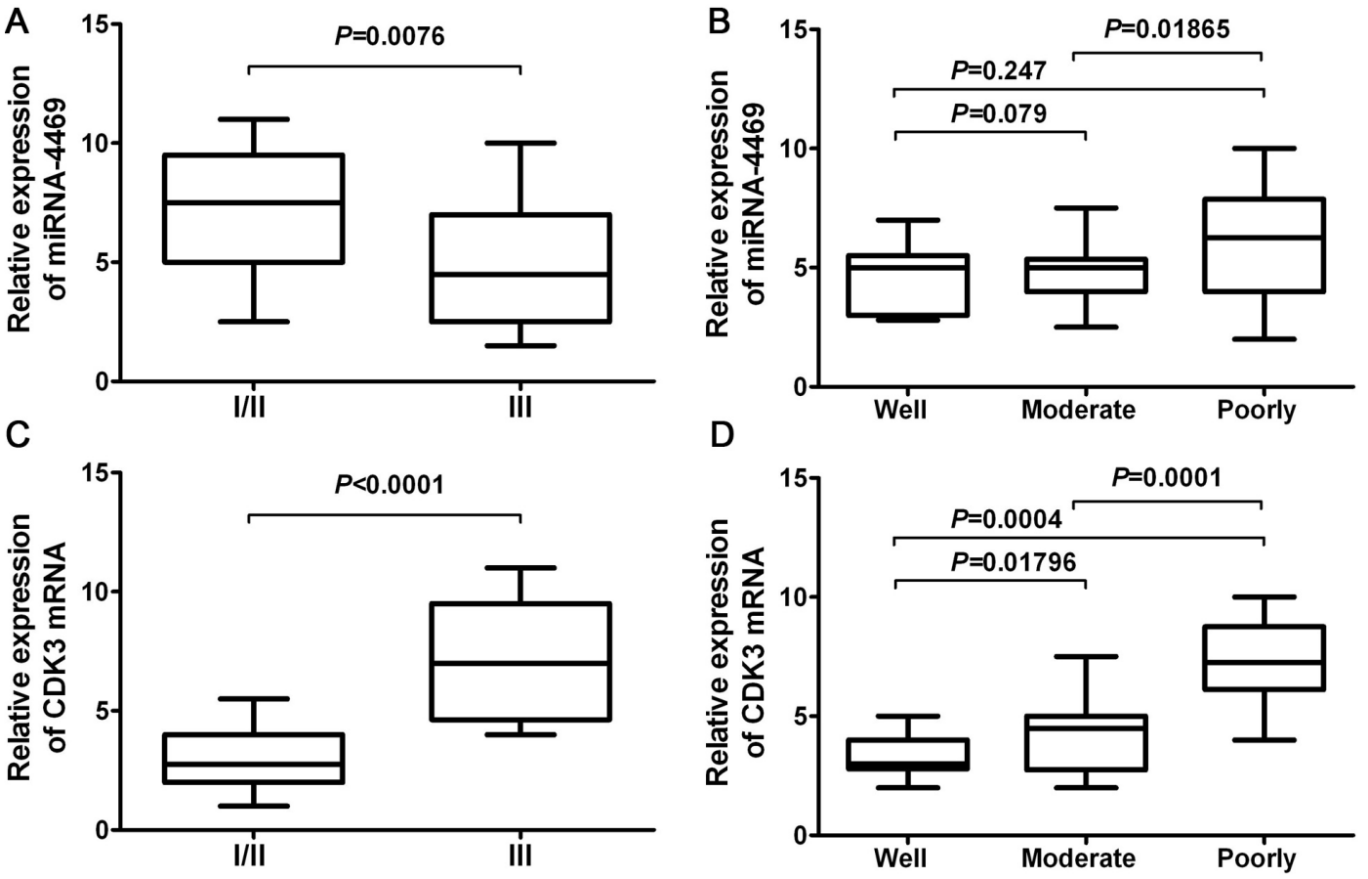
**miR-4469 and CDK3 were both associated with clinicopathological characteristics in ESCC.** (**A**) The association of miR-4469 with TNM stage, (**B**) The association of miR-4469 with histological grade, (**C**) The association of CDK3 with TNM stage, and (**D**) The association of CDK3 with histological grade.

A chi-square test was used to estimate the relationship between miR-4469 and CDK3 expression and the clinicopathological characteristics of patients with ESCC. The results demonstrated that miR-4469 was negatively associated with TNM stage (Figure 7A; P<0.001). However, no significant association was determined between histological grade (Figure 7B; P>0.05). Furthermore, it was determined that CDK3 expression was positively associated with TNM stage (Figure 7C; P<0.001) and negatively associated with histological grade (Figure 7D; P<0.001).

### Rescue assays

**Figure 8:**
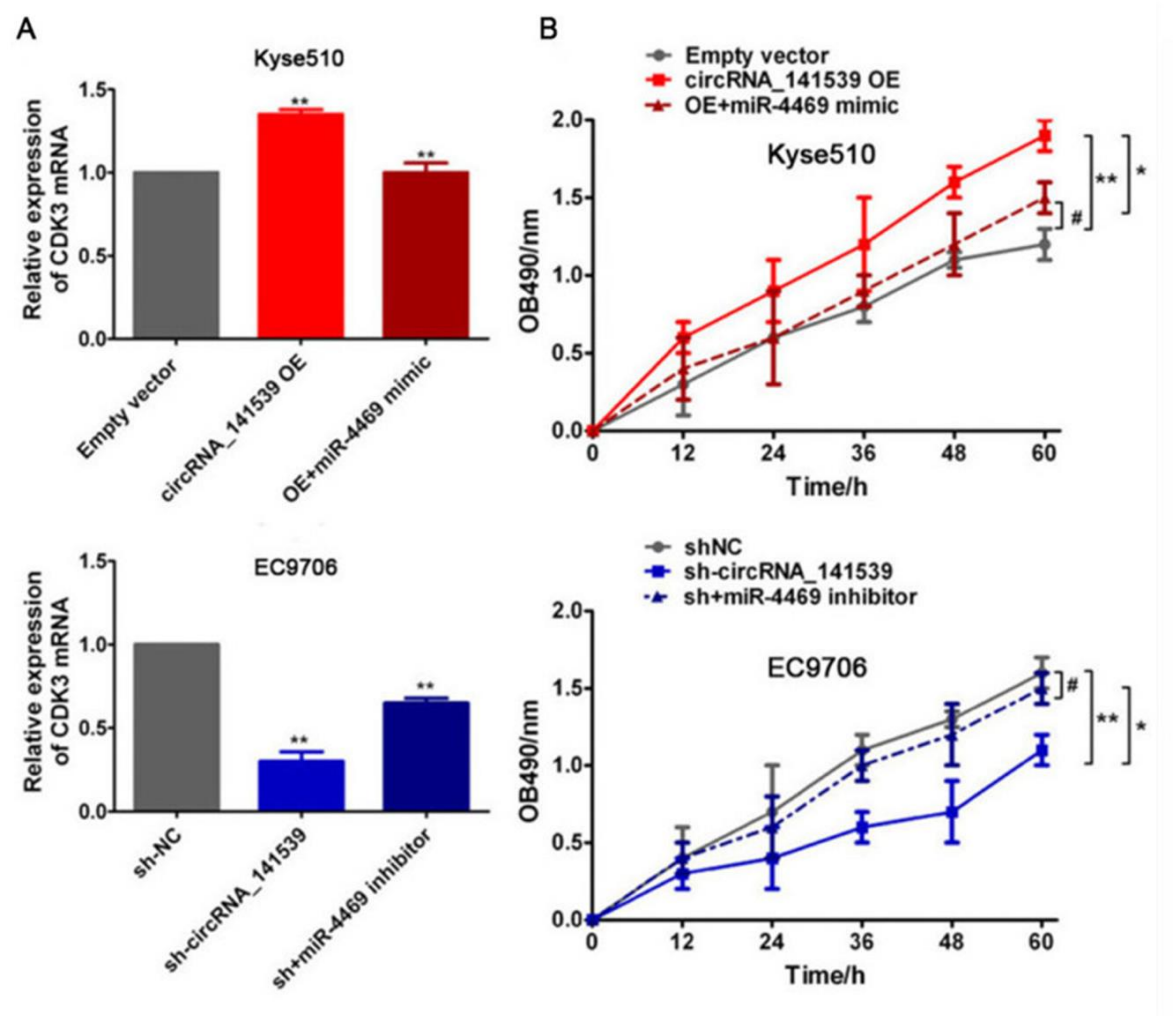
**Rescue experiment, miR-4469 reversed the effects of circRNA_141539 on CDK3 expression and cell proliferation.** (**A**) qRT-PCR assay showed the effects of circRNA_141539/miR-4469 axis on CDK3 mRNA expression. ^*^P < 0.05, ^**^P < 0.01 versus corresponding NC group. (**B**) MTT assay showed the effects of circRNA_141539/miR-4469 axis on cell proliferation. ^*^P < 0.05, ^**^P < 0.01 versus corresponding NC group.

Rescue assays were performed to assess the relationship between circRNA_141539, miR-4469 and CDK3. Kyse510 cells were co-transfected with the circRNA_141539 OE vector and miR-4469 mimics. EC9706 cells were also co-transfected with sh-circRNA_141539 and miR-4469 inhibitors. A subsequent RT-qPCR assay revealed that circRNA_141539 overexpression promoted CDK3 mRNA expression and cell proliferation. Furthermore, circRNA_141539 silencing inhibited CDK3 mRNA expression and cell proliferation, but this effect was reversed following miR-4469 mimic and miR-4469 mimic inhibitor treatment (Figure 8A, 8B; P<0.001).

## DISCUSSION

CircRNA is characterized by a covalent closed loop that lacks 5’ caps and 3’ tails, which is conserved and stably expressed in mammalian cells. Recent studies [11–13] have revealed that circRNAs are abnormally expressed and serve important roles in the development and progression of cancer, serving as potential diagnostic biomarkers and therapeutic targets for patients. Additionally, the function of the circRNAs-miRNA-gene axis in the development and progression of various types of cancer is being elucidated. For example, the circ_0016788/miR-486/CDK4 axis regulates hepatocellular carcinoma tumorigenesis [14, 15]. Furthermore, the circRNA_0084043/miR-153-3p/Snail axis promotes malignant melanoma progression [16, 17] and the circRNA-000911/miR-449a/Notch1 axis promotes chemotherapy resistance in breast cancer [18, 19].

To the best of our knowledge, the present study was the first to assess circRNA_141539 in the occurrence and development of ESCC. Very limited data regarding circRNA_141539 is available. However, Liu et al [20] reported that circRNA_141539 was significantly up-regulated in cervical carcinoma and was correlated with FIGO stage and pathological grade. In the present study, the results revealed that circRNA_141539 was up-regulated and acted as an oncogenic factor in ESCC. Furthermore, high circRNA_141539 expressions were significantly associated with TNM stage, differentiation and poor prognosis, and exhibited high diagnostic value in patients with ESCC. The results indicated that circRNA_141539 may serve as a biomarker for the diagnosis and prognosis of patients with ESCC. Although the sample size of the current study included >50 tissues, large-scale clinical validation is still required. Also, *in vivo* experiments are fundamental as a mandatory step of the bench work to achieve a better correlation with the clinical implications, so further validation *in vitro* was also required in the future.

It is well known that circRNA affects miRNA-target gene expression. The results of the dual-luciferase reporter assay performed in the current study identified that circRNA_141539 directly binds to miR-4469 and that CDK3 is negatively regulated by miR-4469. A previous study [21] reported that miR-4469 was significantly down-regulated in breast cancer and that miR-4469 suppressed breast cancer metastasis by inhibiting the Wnt/β-catenin pathway. CDK3 is a member of the cyclin-dependent kinase family and is directly involved in cell cycle regulation for G1 exit and S entry [22, 23]. The activation of CDK3 leads to G1 arrest and serves an important role in cell proliferation and malignant transformation [24]. CDK3 is abnormally expressed in several types of cancer, including gastric cancer, lung cancer, liver cancer, pancreatic cancer, colorectal cancer, head and neck squamous cell carcinoma, cervical cancer and breast cancer [25]. Furthermore, CDK3 expression may be regulated by certain miRNAs in a variety of different cancers, including miR-873 [26], miR-125a-3p [27] and miR-150 [28]. Also, there are some other factors that can regulate CDK3 expression such as NFAT3, pRb and Ik3-2 gene. MicroRNAs are only one of the important parts of the regulatory mechanism of CDK3.The results of the present study indicated that miR-4469 was down-regulated and CDK3 was up-regulated in ESCC tissue. In addition, miR-4469 was associated with TNM stage and CDK3 expression was associated with TNM stage and histological grade in patients with ESCC. The rescue assays performed in the current study indicated that miR-4469 reversed the effects of circRNA_141539 on cell proliferation. The results indicated that circRNA_141539 promoted ESCC progression via the miR-4469/CDK3 pathway. However, the underlying mechanism still requires further elucidation.

## CONCLUSIONS

In summary and to the best of our knowledge, the current study was the first to determine that circRNA_141539 was significantly up-regulated in patients with ESCC and that high circRNA_141539 expressions were significantly associated with TNM stage, differentiation and a poor prognosis. Additionally, circRNA_141539 was determined to have a high diagnostic value. CircRNA_141539 may serve as an oncogenic factor for patients with ESCC by sponging miR-4469 and activating the CDK3 gene. The circRNA_141539/miR-4469/CDK3 axis may represent a novel diagnostic biomarker, prognostic biomarker and therapeutic target for patients with patients. However, large-scale clinical validations and further mechanistic explorations are still required.

## MATERIALS AND METHODS

### Materials

### Clinical samples and cell lines

ESCC tissues and adjacent non-cancer tissues (50 pairs) were obtained from patients admitted to the First Affiliated Hospital of China Medical University from February 2016 to August 2017. All tumor samples were obtained following surgery and stored in liquid nitrogen at -80° C until further use. Additionally, all samples were confirmed via histopathology and the clinicopathological features of tissue were determined (Supplementary Table 2). Patient follow-up started from the date of surgery until the disease procession or the last follow-up (median follow up period, 23 months, the maximum follow-up time, 31months). In addition, the following human ESCC cell lines were purchased from the Cell Bank of Type Collection of the Chinese Academy of Sciences: Kyse410, Kyse510, EC9706, ECA109 TE7 and Het-1A. All experiments performed in the study current were approved by the Hospital Ethics Committee.

### Reagents and instruments

RPMI1640, fetal bovine serum, MTT, DMSO, crystal violet, the PCR reverse transcription kit and the Transwell chamber kit were all purchased from Invitrogen (Thermo Fisher Scientific, Inc.). Anti-rabbit CDK3 primary antibodies (1:500; cat. no. sc-81836) and horseradish peroxidase (HRP)-conjugated secondary antibodies (1:1,000; cat. no. R2655) were purchased from Gibco; Thermo Fisher Scientific, Inc. and Sigma-Aldrich; Merck KGaA, respectively. Arraystar Human circRNA chip (ArrayStar; Sigma-Aldrich; Merck KGaA) detection and data analysis were performed by GeneChem, Inc. Luciferase reporter gene vectors were also synthesized and constructed by GeneChem, Inc. miR-4469 negative controls (NCs), mimics and inhibitors were synthesized and further transfected and constructed by GeneChem, Inc.

### Methods

### RNA extraction and circRNA chip detection

Total RNA was extracted using the RNeasy Mini kit (Qiagen GmbH). CircRNAs were subsequently enriched and linear RNAs were removed using the Rnase R. Arraystar Human circRNA chip (8x15 K; Arraystar, Inc.). The aforementioned chip was also used to identify differentially expressed circRNAs (three pairs of samples). CircRNA chip detection and data analysis were performed by GeneChem, Inc. The GEO accession number was GSE97332.

### Reverse transcription-quantitative PCR (RT-qPCR)

Total RNA was isolated using the TRIzol^®^ reagent. The forward (F) and reverse (R) primers utilized in RT-qPCR were designed and synthesized by GeneChem, Inc. The sequences were as follows: circRNA_141539 F, 5’-CAGCTGTGACAGCATGATGA-3’ and R, 5’-TCGGGCAT CACCCGAAACAA-3’; miR-4469 F, 5’-GAATTCATCTCGACACGC-3’ and R, 5’-A CTCG GCAG CACAGACAG-3’; CDK3 F, 5’-CCAGCTCTTTCGTATCTTTCGT-3’ and R, 5’-TTCCTGGTCCACTTAGGGAAG-3’. RNA was reverse transcribed into cDNA using the High-Capacity Reverse Transcription kit and RT-qPCR was performed with the SYBR Green PCR Master mix system. Relative expression was calculated using the 2^−Δ∆Ct^ method. RNA levels were normalized to GAPDH (for circRNA_141539 and CDK3 mRNA) or U6 (for miR-4469).

### Cell transfection

Plasmid-mediated circRNA_141539 overexpression (full-length DNA sequence clone), vectors (circRNA_141539 OE) and short interfering RNA (shRNA) targeting circRNA_141539 (sh-circRNA_141539) were designed using the circinteractome tool (https://circinteractome.nia.nih.gov/bin/), synthesized and inserted into pcDNA3.1 vectors and HBLV-U6 lentivirus vectors by GeneChem, Inc. pcDNA3.1 empty vectors and sh-NCs were used as NCs. Restriction enzyme sites for EcoRI and BamHI were used to clone circRNA_141539. The concentration of sh-circRNA_141539 and circRNA_141539 OE used for transfection was 50 nM. miR-4469 mimics, miR-4469 inhibitors and the NC were also synthesized and constructed by GeneChem, Inc. Cells were transiently transfected using Lipofectamine^®^ 2000 (Invitrogen; Thermo Fisher Scientific, Inc.), after which transfection efficiency was verified via RT-qPCR.

### MTT assay

Transfected ESCC Kyse510 and ECA109 cells in the logarithmic growth phase were seeded into 96-well culture plates and incubated for 0, 12, 24, 48 and 72 h at 37° C with 5% CO_2_. Following culture for various durations, MTT solution (20 μl; 2.5mg/ml) was added to each well for 4 h. Subsequently, dimethyl sulfoxide (100 μl) was added to each well to dissolve the resulting crystals. Absorbance values at 490 nm (OB490 nm) were measured using an FL600 microtiter plate reader.

### Transwell invasion assay

Transfected ESCC Kyse510 and ECA109 cells in the logarithmic growth phase were digested, counted and seeded into the upper membrane of a Transwell chamber. The lower chamber was coated with fibronectin (1 mg/ml) and medium containing 20% fetal bovine serum. After culture for 24 h, cells that passed through the fibronectin layer were fixed with 2% paraformaldehyde and stained with crystal violet. Stained cells (invading Kyse510 and ECA109 cells) were subsequently observed and imaged under an Olympus microscope (magnification, x400). The number of stained cells was counted with ImageJ 1.47 software (National Institutes of Health) in five random fields of view.

### Luciferase assay

Wild-type (Wt; circRNA_141539-Wt and CDK3-Wt) or mutant (mu; circRNA_141539-Mu and CDK3-Mu) psiCHECK2 plasmids were designed and synthesized by GeneChem, Inc. Plasmids were co-transfected into Kyse510 and ECA109 cells with miR-4469 mimics and miR-4469 NC according to the manufacturer’s protocol. Luciferase assays were performed using a dual-luciferase reporter gene assay kit. Mu plasmids were used as controls. Luciferase activity was measured using the dual-luciferase reporter gene assay system and Renilla luciferase activity was normalized against that of firefly luciferase. The aforementioned processes were conducted by GeneChem, Inc.

### Western blotting

Total protein of transfected ESCC Kyse510 and ECA109 cells in the logarithmic growth phase was obtained using RIPA buffer. Total protein concentrations were subsequently quantified using a BCA Protein assay kit. Isolated protein (50 μg) were separated via 10% SDS-PAGE, transferred to polyvinylidene difluoride membranes, blocked with 5% non-fat milk and incubated with primary antibodies against CDK3 (1:500; cat. no. sc-81836-1; Gibco; Thermo Fisher Scientific, Inc.) overnight. Samples were then incubated with HRP-conjugated secondary antibodies (1:500; cat. no. c-81836-2; Gibco; Thermo Fisher Scientific, Inc.). Resultant bands were visualized using an ECL-PLUS kit and analyzed using Image J 1.47 software.

### Statistical analysis

SPSS17.0 statistical software (version 7.0) and GraphPad Prism (version 5.01) were used for statistical analysis. Experiments were repeated at least three times and data are presented as the mean ± standard deviation (x±s; α=0.05). The chi-square test, Kaplan-Meier method and Multivariate Cox analysis were performed to investigate the association between circRNA_141539 expression and the clinicopathologic characteristics and prognosis of patients with ESCC. Paired and independent t-tests or rank-sum tests were applied when only two groups were compared, whereas comparisons among multiple groups were analyzed via one-way analysis of variance followed by Tukey's multiple comparison test. P<0.05 was considered to indicate a statistically significant difference.

## Supplementary Material

Supplementary Figures

Supplementary Tables
