# Supplementary material for: circRNA_141539 can serve as an oncogenic factor in esophageal squamous cell carcinoma by sponging miR-4469 and activating CDK3 gene
Source: Aging (Albany NY). 2021 Jan 27;13(8):12179–93. doi: 10.18632/aging.103071 (PMC8109109; doi:10.18632/aging.103071)
Supplement: Supplementary Figures [file aging-13-103071-s001.pdf]

SUPPLEMENTARY FIGURES

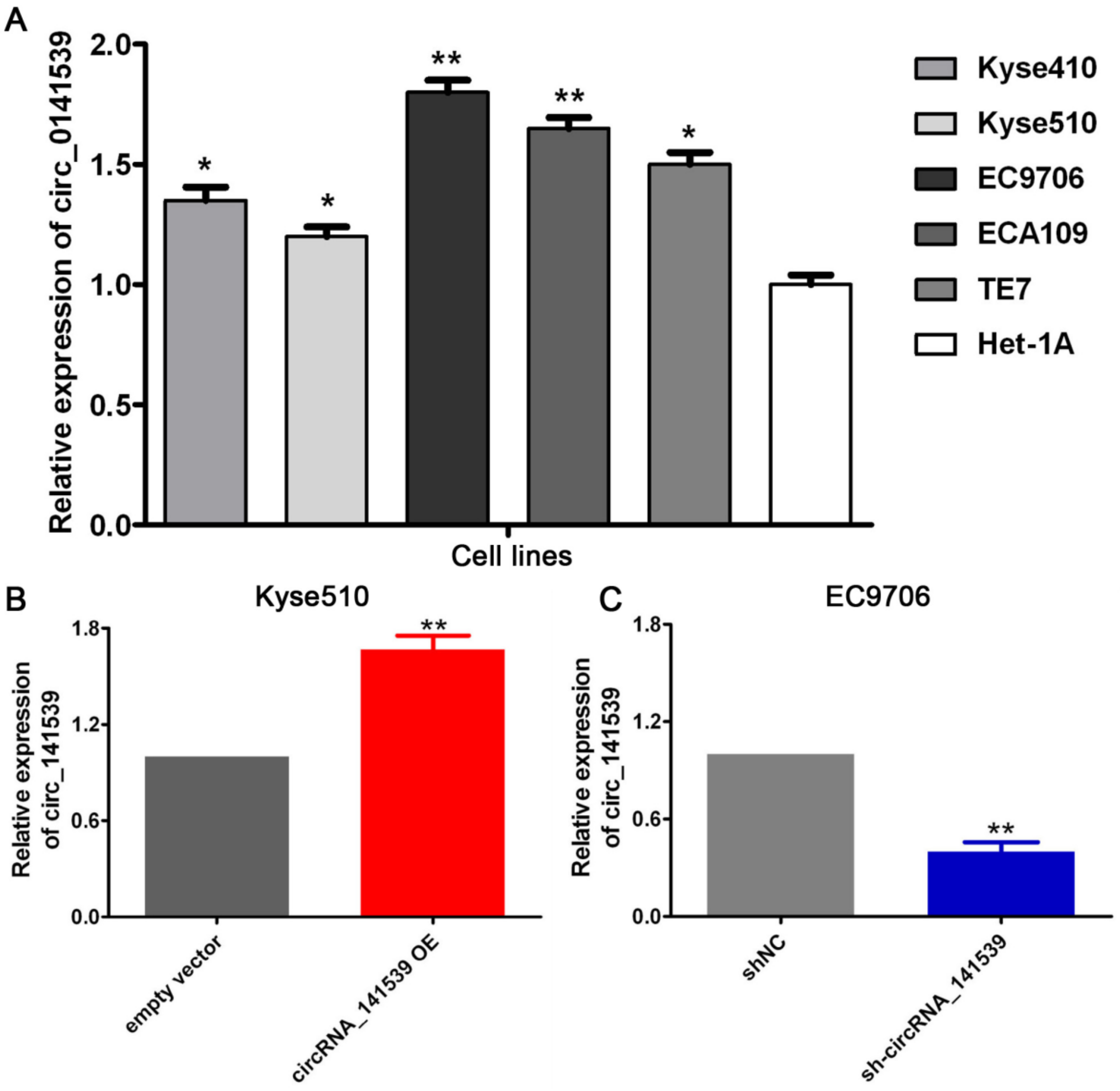

Supplementary Figure 1. circRNA_141539 was up-regulated in ESCC cells and cell transfection. (**A**) qRT-PCR assay showed that circRNA_001937 was significantly up-regulated in ESCC cells, $^{*}P < 0.05$, $^{**}P < 0.01$ versus Het-1A cells. (**B, C**) Transfection efficiency were verified by qRT-PCR. $^{*}P < 0.05$, $^{**}P < 0.01$ versus corresponding NC group.

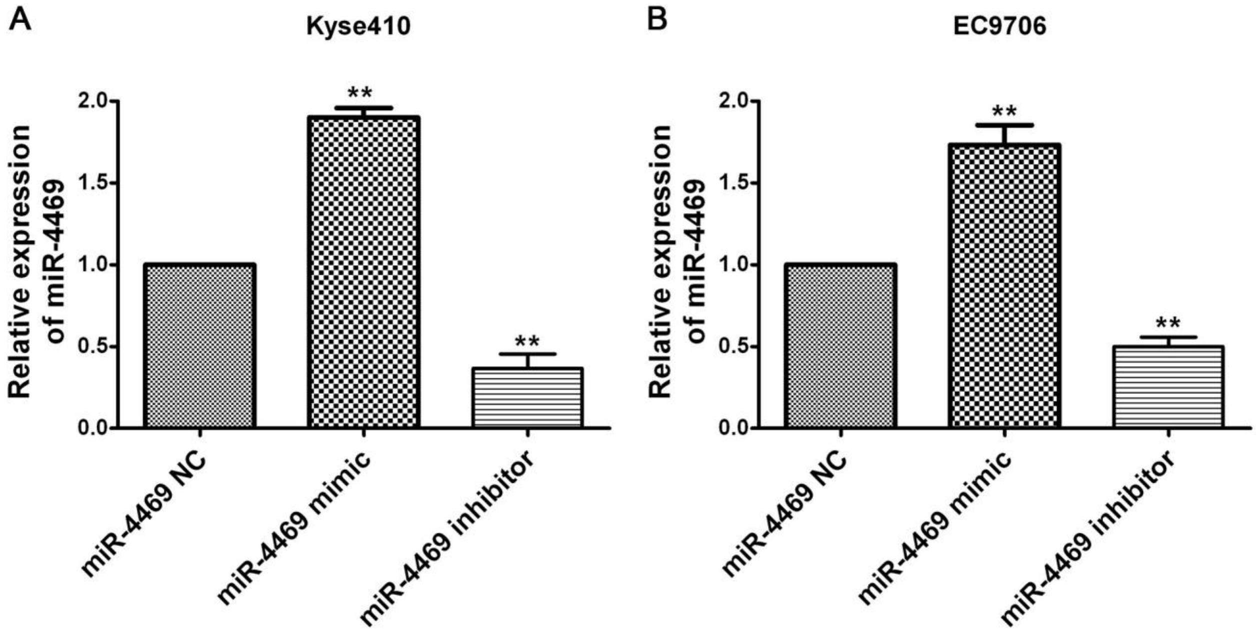

Supplementary Figure 2. Transfection efficiency of miR-4469 as determined via reverse transcription-quantitative PCR. (**A**) Kyse510 cells, (**B**) EC9706 cells. $^{*}P < 0.05$, $^{**}P < 0.01$ versus corresponding NC group.
