# Supplementary material for: circRNA_141539 can serve as an oncogenic factor in esophageal squamous cell carcinoma by sponging miR-4469 and activating CDK3 gene
Source: Aging (Albany NY). 2021 Jan 27;13(8):12179–93. doi: 10.18632/aging.103071 (PMC8109109; doi:10.18632/aging.103071)
Supplement: Supplementary Tables [file aging-13-103071-s002.pdf]

SUPPLEMENTARY TABLES

Supplementary Table 1. The top 10 significantly up/down -regulated circRNAs.

circRNA	P-value	FC	Regulation	Source	Chrom	Type	GeneSymbol
hsa_circRNA_035855	0.03615	7.64374	up	circBase	chr15	exonic	PIF1
hsa_circRNA_400075	0.01663	7.60343	up	circBase	chr3	intronic	RPL29
hsa_circRNA_141539	0.02044	7.58017	up	25070500	chr6	intronic	C6orf106
hsa_circRNA_101337	0.00195	6.93262	up	circBase	chr14	exonic	HEATR5A
hsa_circRNA_100882	0.04987	6.12234	up	circBase	chr11	exonic	STARD10
hsa_circRNA_031267	0.00767	5.91116	up	circBase	chr14	exonic	PSMB5
hsa_circRNA_407088	0.04430	5.20779	up	25070500	chr8	exonic	EYA1
hsa_circRNA_051241	0.02310	4.90222	up	circBase	chr19	exonic	RPS19
hsa_circRNA_016458	0.02619	4.89278	up	circBase	chr1	exonic	KCNK2
hsa_circRNA_008410	0.03107	4.59272	up	circBase	chr17	intronic	PGS1
hsa_circRNA_405241	0.01602	9.10119	down	25070500	chr14	exonic	PPM1A
hsa_circRNA_091722	0.03101	9.09461	down	circBase	chrX	exonic	MAGEA3
hsa_circRNA_404987	0.01651	8.79459	down	25070500	chr12	overlapping	ERGIC2
hsa_circRNA_100696	0.03132	8.08075	down	circBase	chr10	exonic	PPAPDC1A
hsa_circRNA_001015	0.04874	7.97024	down	circBase	chr2	intronic	USP34
hsa_circRNA_406058	0.02140	7.06160	down	25070500	chr2	intronic	RNU2-22P
hsa_circRNA_003613	0.03224	6.83637	down	circBase	chr1	exonic	NRD1
hsa_circRNA_089652	0.02254	6.12656	down	circBase	chr9	exonic	TPRN
hsa_circRNA_100899	0.02291	5.81777	down	circBase	chr11	exonic	INTS4
hsa_circRNA_102832	0.04090	5.61607	down	circBase	chr2	exonic	ARL6IP6

FC, fold change.

Supplementary Table 2. Patients' clinicopathologic characteristics.

Characteristics	Cases (n)
Median age (years)	68 y
Gender	
Male	35
Female	15
Pathological types	
Adenocarcinoma	0
Squamous cell carcinoma	50
TNM stage	
I/II	12
III	38
Pathological stage	
High and middle	19
Low	21
